# Development of an Acid-Protective Polymer Encapsulation Formulation for Oral Delivery of Salmonella Phages

**DOI:** 10.3390/v17091205

**Published:** 2025-09-02

**Authors:** Manju Bernela, Nitin Virmani, Bidhan Chand Bera, Rajesh Kumar Vaid, Medhavi Vashisth, Taruna Anand

**Affiliations:** 1 ICAR-National Research Centre on Equines, Hisar 125001, Haryana, India; manju.barnela@gmail.com (M.B.); nvirmani@gmail.com (N.V.); 2 National Centre for Veterinary Type Cultures, ICAR-National Research Centre on Equines, Hisar 125001, Haryana, India; bcbpatent@gmail.com (B.C.B.); rk_vaid@yahoo.com (R.K.V.); medhavi3868@gmail.com (M.V.)

**Keywords:** *Salmonella*, phage, oral delivery system, poultry

## Abstract

Bacteriophage therapy can successfully provide additional treatment to control *Salmonella* infection, but low gastric pH limits its oral application. The present study aimed to develop an improved encapsulation formulation with enhanced acid protection for oral delivery of *Salmonella* phages using polymers. This was achieved by encapsulating a phage cocktail containing three different bacteriophages against *Salmonella* sp. in alginate beads incorporating polyvinyl alcohol (PVA), PVP-K30, and calcium carbonate as viscosity modifiers and acid protection enhancers. Further, the beads were coated with poly-L-lysine to improve the stability and tested for their efficacy for improved phage viability under in vitro acidic conditions for subsequent use in oral delivery. Moist beads were slimy, and semi-dried beads presented a coarse surface as observed using FE-SEM. *In vitro* studies revealed that the free phage cocktail exhibited complete inactivation when exposed to acidic pH 2.5 after 15 min incubation. In contrast, the encapsulated phage cocktail showed a decrease of only 1.66 log units in viability when incubated for 90 min at pH 2.5. Furthermore, oral delivery of the encapsulated phage cocktail in the poultry model significantly reduced bacterial load in infected birds’ intestines.

## 1. Introduction

*Salmonella*, a Gram-negative, rod-shaped bacterium that colonizes various animals’ intestinal tracts, causes enteric diseases associated with diarrhea through contaminated foods [1,2]. There are ~2500 serotypes of *Salmonella* sp. [3]. The pathogenesis of *Salmonella* sp. is attributed to its capability to invade intestinal epithelial cells and survive intracellularly within macrophages [4]. The predominant typhoidal serotypes of *Salmonella enterica* include *S. typhi* and *S. paratyphi*. Still, the leading cause of nontyphoidal *Salmonellae* (NTS) sp. infections exhibiting a broad animal-host range and survival in wider type environments includes *S. typhimurium* and *S. enteritidis* [5,6,7,8,9,10]. The global burden of NTS invasive disease was estimated to be 535,000 cases in the year 2017, particularly affecting children, the elderly, and the immunocompromised [11]. NTS presents a reservoir for resistance determinants, and as such, the surveillance of resistance to critical antimicrobial agents has been a part of monitoring programmes in some countries [12,13]. However, the infections caused by NTS are not easily controlled by antibiotics since natural selection gives bacteria the upper hand after decades of antibiotic usage. Evidence includes the outbreaks of *S. typhimurium* that have occurred worldwide, the majority of which were caused by multidrug resistant *S. typhimurium* [14,15,16], posing difficulties for clinical treatment, resulting in increased morbidity and mortality [17]. A high prevalence of resistance to colistin, nalidixic acid, sulfamethoxazole, tetracycline, cephalosporin, and quinolone has also been reported in *S. enterica ser.* Enteritidis [18,19] in the recent past. Increasing cases of antibiotics becoming ineffective, coupled with a decline in the development and production of novel antibiotics, present a global issue of growing concern that could lead to increasingly serious health consequences [20,21].

Thus, alternatives need to be identified urgently. The revitalization of phage therapy is believed to be one viable solution to the antimicrobial resistance crisis [22]. Bacteriophages are viruses that can invade and destroy bacteria in human or animal cells with no adverse effects and are intended to treat bacterial infections. The most appealing characteristic of phages is their precision of action, eliminating the possibility of infecting beneficial bacteria [23]. Increasing acceptance of bacteriophage therapeutics is apparent from FDA approval for the first clinical study for Phage Bank to treat multidrug resistant bacteria [24]. However, oral phage delivery suffers limitations such as phage inactivation due to the acidic and proteolytic environment of the stomach [25]. Given this, phage encapsulation strategies have been adopted to provide a safe phage delivery technique with minimal losses [26,27]. Polymers have always been a material of choice for designing phage delivery systems. Alginate beads are considered a good candidate for phage encapsulation due to their ability to resist acidity and regulate and maintain the release of live products to the gut [28,29]. Still, poor retention of hydrophilic entities, which tend to escape during the bead preparation process, poses a problem. Moreover, alginate beads have been reported to provide partial protection against gastric conditions [30]. Co-encapsulation of calcium carbonate (CaCO_3_) in beads improved the phage stability in simulated gastric fluids [31]. The leaching effect can be minimized by the incorporation of polyvinyl alcohol (PVA) into the alginate solution and polyvinyl pyrrolidone (PVP) into the CaCl_2_ solution, where PVA works as a viscosity modifier and PVP acts as a pore blocker [32,33]. Accordingly, the objective of the current study was to prepare phage-loaded alginate beads while incorporating all these factors to obtain optimum performance of encapsulated phages against *Salmonella* sp. when administered via the oral route. We envisaged that the incorporation of CaCO_3_, PVA, and PVP as excipients into the Ca-alginate macrospheres may strengthen the alginate gel network and buffer the acidic intra-particle pH, thus enhancing the acid protection of the encapsulated phage. Poly-L-lysine was evaluated as a coating material to improve the stability. The developed delivery system was further evaluated in a poultry model for efficacy evaluation.

## 2. Materials and Methods

### 2.1. Animals

One hundred and thirty broiler chickens were procured from Disease Free Small Animal House, LUVAS, Hisar, and kept for 10 days in the Animal House for acclimatization to the new environment. Prior permission from the Institutional Animal Ethical Committee (IAEC) of the ICAR-NRCE was received via letter no. NRCE/CPCSEA/2018-19 dated 7 February 2019.

### 2.2. Bacterial Strains and Culture Conditions

The bacterial strains were *Salmonella typhimurium* (VTCCBAA702), *Salmonella enteritidis* (VTCCBAA722), and *Salmonella enterica* (VTCCBAA714) obtained from the Bacterial Repository of National Centre for Veterinary Type Cultures, ICAR-NRCE, Hisar, Haryana, India.

### 2.3. Bacteriophage Isolation and Characterization

#### 2.3.1. Phage Enrichment and Spot Assay

Bacteriophages against salmonellae were isolated from poultry litter and sewage samples. Briefly, phage enrichment was carried out using one gram of litter sample suspended in 40 mL PBS and mixing with 5 mL of 5x nutrient broth (NB) + 5 mL of exponentially grown host bacterial culture and incubating under shaking conditions at 37 °C overnight. After incubation, centrifugation was carried out at 10,000 rpm for 15 min, followed by filtration (0.22-µm PVDF membrane filter). The bacteriophage activity was detected in the filtrate by spotting 5 µL of the filtrate on nutrient agar (NA) seeded with host bacteria. The development of a clear lytic zone indicated phage-mediated bactericidal activity. Single plaque in the filtrate was purified by plaque formation assay by picking thrice in SM buffer (5.8 g/L NaCl, 2.0 g/L MgSO_4_, 50 mL/L 1M Tris, pH 7.5, 5 mL/L pre-sterilized 2% *w*/*v* gelatin) and replating. The plaque characteristics and phage titer were determined. The bacteriophage plaques were observed using the double-layer agar method [34]. Briefly, sterile 1.5% nutrient agar was poured in a 90 mm Petri plate. Then bacteriophage suspension (100 uL) was mixed with early log-phase bacterial culture (300 uL) and this suspension was further mixed in sterile molten 0.75% agar. This mix was then poured over the agar plate and was allowed to dry. The Petri plates were then incubated overnight at 37 °C and the clear zone of lysis, representing distinct bacteriophage plaques, were observed, and their morphology was recorded in terms of their shape, size, and appearance.

#### 2.3.2. Bulk Propagation of Bacteriophages

A total of 10^9^ PFU of purified phage suspension was mixed with the bacterial pellet obtained from 2 mL culture and co-incubated for 10 min to allow phage adsorption, suspended in 50 mL NB, and incubated under shaking conditions for 8–12 h at 37 °C. Chloroform (10 mL) was added, and the suspension was centrifuged at 8300 rpm for 10 min. It was treated with pancreatic Dnase I and Rnase (1 mg/mL) for 30 min, and NaCl (1 M) and PEG 8000 (10% *w*/*v*) were added to the suspension and centrifuged to obtain the phage pellet, which was then dissolved in SM buffer. It was re-purified by chloroform (1:1 *v*/*v*) extraction.

#### 2.3.3. Assessment of Thermal and pH Sensitivity of Bacteriophages

Assessment of thermal stability of individual bacteriophage suspensions was carried out by incubating separately for 1 h at 4 °C, 25 °C, 37 °C, 45 °C, 55 °C, 65 °C, and 80 °C. Stability assessment at different pH (pH 3 to 10) was carried out by mixing bacteriophage suspensions with sodium acetate/phosphate buffers in the ratio of 1:10 (*v*/*v*) for 1 h, and PFU post-exposure was calculated by the agar overlay method. All assays were performed in triplicate.

#### 2.3.4. Transmission Electron Microscopy (TEM)

Electron microscopy was carried out by placing the phage suspension over formvar/carbon-coated grids to allow binding of bacteriophage. The grids were blotted dry and negatively stained with 2% *w*/*v* uranyl acetate. The grids were washed and visualized on JEOL JEM1011 (Japan) electron microscope at 80 kV accelerating voltage.

### 2.4. Preparation of Alginate Beads

The use of alginate-based carriers for preserving phages designed for food disease management has been extensively studied. When tested under environments imitating the gastrointestinal tract, such a carrier drastically improved the survival rate over free phages [31,35]. Furthermore, this approach had been used previously in the first *in vivo* studies of encapsulated phages [36,37]. In the present study, alginate beads were prepared using the ionotropic gelation method with modification using PVA and PVP in the preparation process. When a phage–polymer dispersion mixture containing phage, sodium alginate, calcium carbonate, and polyvinyl alcohol was added dropwise into CaCl_2_ and PVP K-30 solution, electrostatic interaction between negatively charged carboxylic acid groups on the alginate backbone and positively charged Ca^2+^ ions in the cross-linking solutions resulted in the instant formation of phage-loaded gelled beads. A mixture containing phage (~10^9^ PFU/mL), polyvinyl alcohol (0.5% *w*/*v*), CaCO_3_ (1% *w*/*v*), and sodium alginate (2% *w*/*v*) was added dropwise (0.5-0.6 mL/min) using an insulin syringe in a bath containing CaCl_2_ (1% *w*/*v*) and Polyvinylpyrrolidone (PVP) K-30 (2% *w*/*v*) on magnetic stirring at a stirring rate of (100 rpm). Phage alginate beads were instantly removed and coated with 1% Poly-L-lysine (0.5% *v*/*v*) for 30 min. The beads were stored at 4 °C for further use.

### 2.5. Characterization of Alginate Beads

#### 2.5.1. Bead Size and Morphology Assessment

Bacteriophage-encapsulated beads were investigated using an inverted microscope (Nikon Eclipse Ni, Japan) for their surface appearance, morphology, and size. Bead surface morphology was also visualized using a field emission scanning electron microscope (FE SEM) (JSM-7610F, Jeol, Japan). The beads were semi-dried, placed on a copper stub, and sputter coated with gold for 30 s and visualized in FE-SEM.

#### 2.5.2. Phage-Loading Efficiency

Phage-encapsulated beads were incubated for 10 min in dissolving buffer (50 mM sodium citrate, 0.2 M sodium bicarbonate, and 50 mM Tris-HCl, pH 7.5) with gentle shaking at room temperature [26]. The number of phages released after destruction of beads was calculated using the double-layer agar plate method. Phage titer was measured in PFU per gram of wet alginate beads (average weight of single bead 10.56 ± 0.17 mg), and phage-loading efficiency (PLE) was estimated using the following equation [38]:

$$PLE=\frac{Amount\;of\;phages\;released\;after\;destruction}{Amount\;of\;phages\;initially\;used}\times 100\%$$

### 2.6. Stability of Beads at Different pH

Stability of free and bead-encapsulated phages were studied in 0.85% (*w*/*v*) NaCl solution with pH adjusted to 2.5/7.4. One hundred µL of phage suspension (10^8^ PFU/mL) or 30 alginate beads (1.1 × 10^6^ PFU/mL) were kept in vials with 2 mL of pH 2.5/7.4 solution at 37 °C. Samples in vials were collected after 15, 30, 45, 60, and 90 min of incubation. Free phage samples were diluted and analyzed immediately for phage survival. In case of beads, beads were collected, rinsed with distilled water, and then dissolved in 1 mL of dissolving buffer, diluted, and assayed for active phages.

### 2.7. Stability of Free and Encapsulated Phage in Bile Salts

**T**o determine the stability of free and encapsulated phages in the presence of bile salt, 100 μL (1.1 × 10^8^PFU) of phage suspensions and phage-encapsulated alginate beads (30) (1.1 × 10^6^ PFU) were incubated with 1% (*w*/*v*) bile extract at 37 °C. Samples were withdrawn after 1 h and 3 h of incubation and mixed with SM buffer, and the phage titer was determined by the double-layer agar method.

### 2.8. Release at pH 7.4

Release of bead-encapsulated phages was investigated in pH 7.4 buffer. Fresh alginate beads (30) were placed into 50 mL of pH 7.4 buffer at shaking (37 °C), and 100 μL of aliquots were withdrawn and replaced with the same amount of buffer at selected time intervals. Samples were titrated in triplicate using the double-layer agar method.

### 2.9. Storage Stability

To determine the stability of beads during the storage period at 4 °C, beads were checked for lytic activity at different time intervals.

### 2.10. In Vivo Evaluation of Polymer-Encapsulated Formulation in Broiler Chickens

To test how well encapsulated bacteriophages protect against *Salmonella typhimurium* and *S. paratyphi* in chickens, the chicks were divided into five groups (30 birds per group, except for the negative control group, which had 10 birds):

Group A: Healthy birds with no infection or treatment (negative control).
Group B: Birds infected with *S. typhimurium* and treated with phage-loaded beads.
Group C: Birds infected with *S. paratyphi* and treated with phage-loaded beads.
Group D: Birds infected with *S. paratyphi* only (positive control).
Group E: Birds infected with *S. typhimurium* only (positive control).

For Groups B and C, we gave the phage bead treatment orally on days 1 and 2 (10 beads per bird, containing 3.6 × 10^5^ PFU). On day 2, these groups were infected orally with 10^5^ CFU of the respective Salmonella strain in 100 mL PBS. After infection, all birds had free access to food and water. Additional phage bead treatments were given after infection on days 3, 4, 6, 9, 11, 14, 16, and 18. The experiment lasted for 24 days. On days 8, 10, 13, 19, and 24, six birds from each group (except control group) were sacrificed for analysis. The poultry birds from all the groups were closely examined for the development of any clinical signs such as mortality, poor general condition, including ruffled feathers, and diarrhea. Bacterial load and phage count were estimated in feces and different organs, including liver, colon, caecum, Ileum, and proventriculus, per gram body weight after trituration in PBS, followed by the spread plate count method. All the experiments were carried out in triplicate to assess the bacterial and phage counts. The experimental data were statistically analyzed using two-way ANOVA followed by Tukey’s multiple comparisons test using Graph Pad Prism version 8.02 software (San Diego, CA, USA). All the findings are shown as mean ± SD (standard deviation). A *p*-value less than 0.05 was set as the basic criterion for the statistical significance of each evaluation.

### 2.11. Histopathological Study

Representative pieces from liver and caecal tonsils were collected during necropsy from broiler chickens. Tissues were immediately fixed in 10% neutral buffered formalin for 24–48 h to preserve cellular architecture and processed for histopathology using standard protocols [39]. Embedded tissues were sectioned at a thickness of 4–5 μm and stained with Hematoxylin and Eosin (H&E) for general histopathological assessment.

### 2.12. Statistical Analysis

All the experiments were carried out in triplicate to assess the bacterial and phage counts. The experimental data were statistically analyzed using two-way ANOVA followed by Tukey’s multiple comparisons test using Graph Pad Prism version 8.02 software (San Diego, CA, USA). All the findings are shown as mean ± SD (standard deviation). A *p*-value less than 0.05 was set as the basic criterion for the statistical significance of each evaluation.

## 3. Results and Discussions

### 3.1. Bacteriophage Isolation

**Figure 1:**
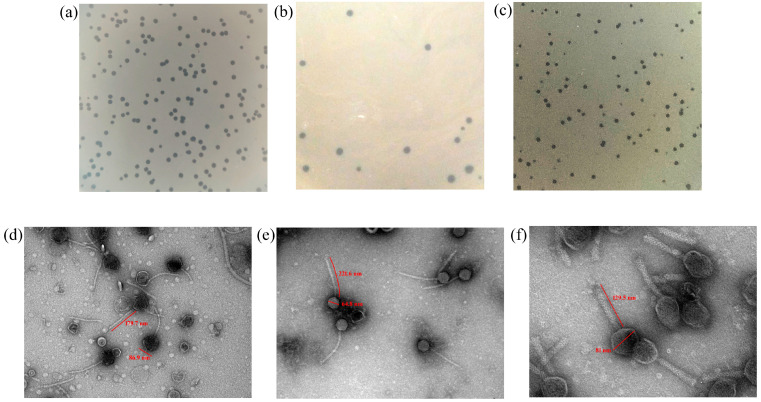
Plaque morphology of (**a**) Salmonella phage ΦST143, (**b**) Salmonella phage ΦST187, and (**c**) Salmonella phage ΦST188 as observed on double-layer agar with host bacterial lawn in a 90 mm Petri dish. Transmission electron microscopic observation of (**d**) Salmonella phage ΦST143 (100,000×), (**e**) Salmonella phage ΦST187 (100,000×), and (**f**) Salmonella phage ΦST188 (200,000×).

The bacteriophages enriched and purified using the indicated host bacteria were observed to produce clear lytic plaques, as shown in Table 1 and in Figure 1a–c.

The phages were observed to be members of the order caudoviricetes by TEM (Figure 1d–f). The capsid diameter and tail length of the three phages, *viz.* ΦST143, ΦST187, and ΦST188 were observed to be 86.9 nm and 179.7 nm; 64.8 nm and 221.6 nm; and 81.0 nm and 129.5 nm, respectively. Temperature and pH stability tests revealed that the enriched phages were active in a temperature range of 4–55 °C and at a pH environment ranging from pH 4 to 9. These three bacteriophages were mixed to prepare a cocktail carrying 10^9^ PFU/mL of each phage and were used further for encapsulation studies.

### 3.2. Preparation of Beads

**Figure 2:**
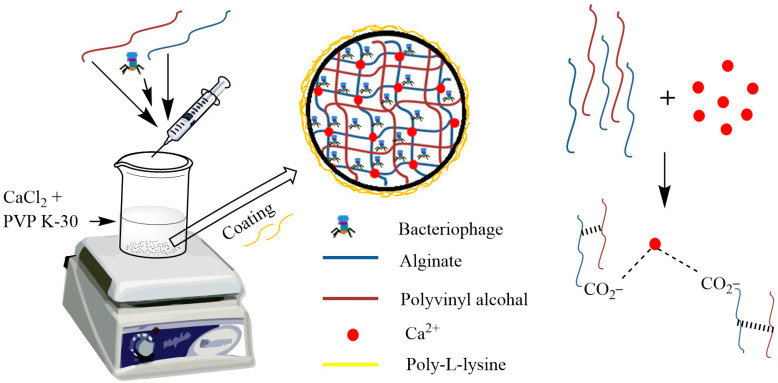
Schematic representation of bead preparation.

The schematic is shown in Figure 2.

The presence of PVA as a viscosity modifier of the alginate solution enhanced the loading and entrapment efficiency of the drug [40]. The addition of PVP increases the viscosity of the cross-linking solution and results in pore blocking of alginate beads, hence increasing the entrapment efficiency of beads [32]. Additionally, it has been reported to enhance circulatory time in plasma when used as a delivery system [41]. In preliminary trials, it was found that the utilization of alginate alone was not able to provide sufficient protection to encapsulate phage; therefore, we modified the bead preparation by employing PVA as well as PVP. These two improved the phage encapsulation efficiency, inhibited the leaching effect, and prevented acid diffusion through alginate pores, consequently providing enhanced acid stability to the encapsulated phage, similar to the reports by Nayak et al., 2011 [42]. The presence of polycations has been shown to have a buffering effect on the surrounding medium layers of microcapsules, which benefits phage safety [26]. Poly-L-lysine was used as a polycation in the present study to strengthen the alginate beads.

### 3.3. Characterization

#### 3.3.1. Bead Size and Morphology

**Figure 3:**
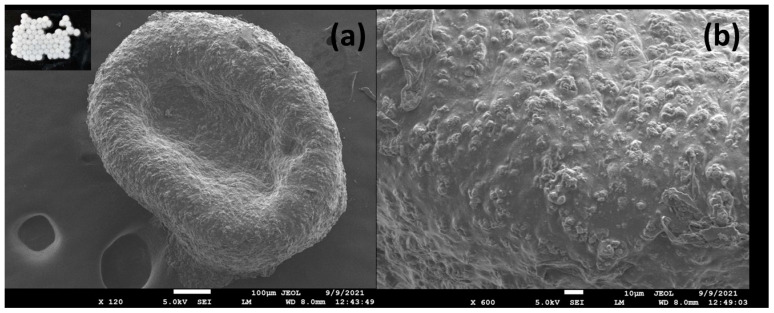
Surface structure of semi-dried alginate beads (**a**) X120, fresh beads (inset), (**b**) as observed by FE-SEM X600.

The size and morphological properties of the phage-encapsulated beads were determined using a scanning electron microscope (SEM). The beads appeared almost spherical, presented a smooth surface, and of uniform size with a mean diameter of 0.8 mm. An image of the phage-alginate beads as observed using an inverted microscope is given in Figure 3 (inset).

The texture of wet beads was spherical with a smooth surface as observed with an inverted microscope (inset Figure 3a), while the texture of dried beads in FE-SEM is presented in Figure 3a,b. Beads were generally spherical with a wrinkled surface and coarse appearance. Because calcium carbonate is poorly soluble in neutral aqueous medium, it exists as tiny particles or fillers in the alginate matrix, resulting in a coarse structure [31].

#### 3.3.2. Phage-Loading Efficiency

The initial phage titer before encapsulation was 9.04 log_10_ PFU/mL. Phage loading at 6.04 log_10_ PFU/g of beads presented a phage encapsulation efficiency of 66.82%. Although 3-log reduction in phage titre was observed during encapsulation, a good entrapment efficiency was achieved in alginate beads.

### 3.4. Stability of Beads at Different pH

**Figure 4:**
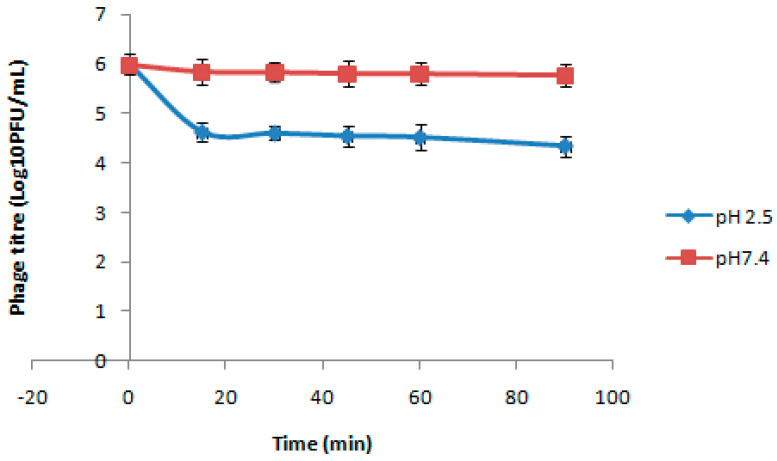
Titers of encapsulated phages after certain incubation periods in different pH.

Stability of the coated beads exposed to acidic pH 2.5 and intestinal pH 7.4 for different time intervals was determined at 37 °C. Bead-encapsulated phages showed viability at the pH indicated for all the tested intervals up to 90 min, although a log reduction in phage count was observed with increasing time intervals following incubation under acidic pH conditions. Figure 4 shows the phage titre for different treatment times at different pH. It can be observed from the figure that encapsulation of bacteriophages in poly-L-lysine-coated alginate beads provided a shielding effect against acidic environment, with a nearly 1.66 log_10_ PFU reduction at pH 2.5, and a complete inactivation of the free phage was observed even after 15 min. In case of pH 7.4, only a 0.2 log_10_ reduction was observed after 90 min of exposure with the encapsulated phage formulation.

This is consistent with a prior study that found no phage in simulated gastric juice after 2 min at pH 2.5 to 2.7 [43]. The alginate gel network shrinks at low pH while swelling at higher pH, which allows both phage protection in gastric conditions and the phage release at intestinal pH in a viable form [26].

### 3.5. Stability of Free and Encapsulated Phage in Bile Salts

The results in Table 2 indicate the effect of bile salts on the survival of free and encapsulated bacteriophages. It can be observed that encapsulation provided significant protection to the bacteriophage against bile salts. The titers of free and encapsulated phage decreased by 3.44 and 0.26 log units, respectively, after 3 h incubation in 1% bile salts, which indicates that the viability of encapsulated phage was highly preserved after 3 h incubation. There are dissimilar reports on the sensitivity of phages to bile salts; for example, *Vibrio vulnificus* phage is highly resistant, whereas the *Escherichia coli* phage T4 is less tolerant [26,43]. The phage cocktail was also sensitive to bile acids, as observed from the results of the present study.

However, Poly-L-lysine-coated alginate beads carrying phages exhibited a high stability against bile salts, as apparent from Table 2. This could be because negatively charged bile acids most likely reacted with polycations on the beads’ surface to produce insoluble complexes. This raises the mass transfer resistance at the surface, inhibiting entry of bile salts into the matrix, hence avoiding phage-destructive effects [26].

### 3.6. Release at pH 7.4

**Figure 5:**
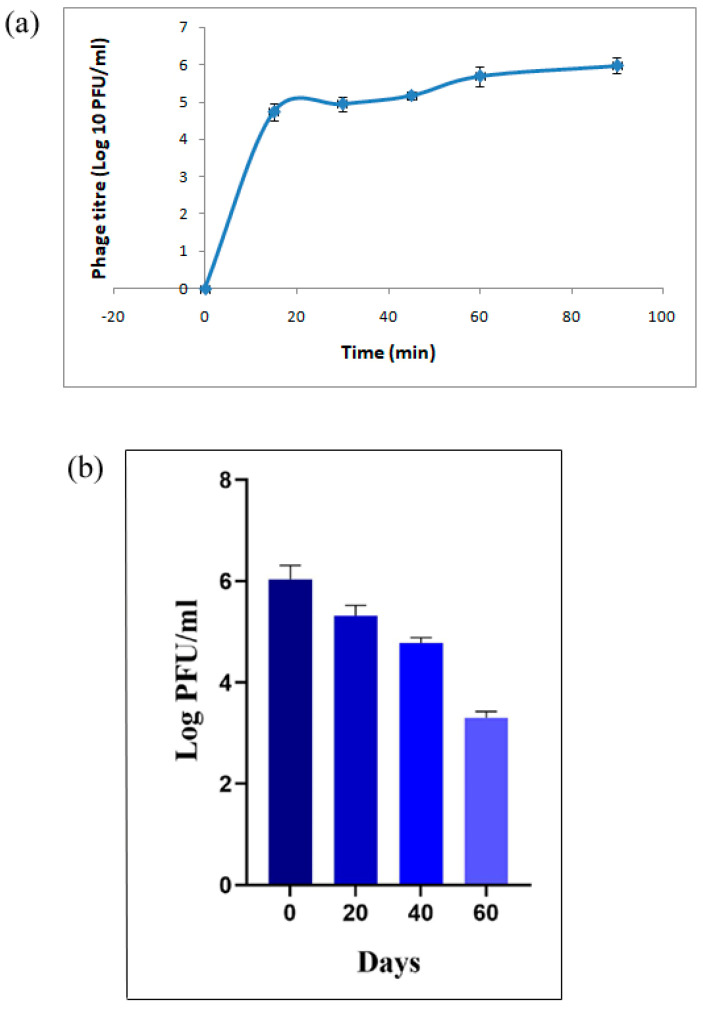
(**a**) Release of encapsulated phage at pH 7.4 at 37 °C. (**b**) Storage stability of phage-cocktail-encapsulated beads after storage.

Macrospheres should not only shield the phage from acidic pH, but also allow the phage to be released in a viable form at the targeted location. The phage release curve for poly-L-lysine-coated phage alginate beads at different periods is presented in Figure 5. When the encapsulated phage was placed in a buffer of pH 7.4, the beads started to swell and disintegrate, giving a burst release effect followed by sustained release. The phage numbers steadily increased from 5.5 × 10^4^ PFU for 15 min to 9.5 × 10^5^ PFU (almost 18% release) for 90 min incubation in pH 7.4 buffer. The release effect of the phage from the alginate matrix is based on a swelling–dissolution–erosion process. When calcium ions attached to alginate molecules are exposed to a pH 7.4 buffer, they start to dissociate through an ion-exchange process with Na^+^ and K^+^ ions, causing the alginate gel to swell and eventually disintegrate [44]. A sustained release effect can be assigned to calcium carbonate particles in the encapsulation matrix because free Ca^2+^ dissociated from calcium carbonate inhibits gel swelling, resulting in the phage release from alginate microspheres [31].

Phages have an icosahedral head (64–87 nm) and a long tail (ranging approximately 130–221 nm), whereas alginate gels are typically nanoporous (up to 5 nm) [45]. This pore size is tiny enough to hold phages until the gel swells to the point where the mesh size is larger than the phage particles. Hence, phage release from alginate beads appears to be mediated by alginate swelling and erosion.

### 3.7. Storage Stability

The storage temperature has a significant impact on stability. Previously published findings showed that phages kept at 4 °C were more stable than those stored at room temperature [28]. So, beads stored at 4 °C were studied for log reduction in phage viability for a period of 60 days (Figure 5b). Log reduction of 3.3 was noticed after 60 days of storage.

### 3.8. In Vivo Evaluation in Broiler Chickens

#### 3.8.1. Assessment of Clinical Signs in Infected Birds

Post bacterial infection, the birds in the groups B, C, D, and E were dull and depressed and developed diarrhea initially, but the intensity of the clinical signs was more pronounced in Group D and E, which was characterized by watery diarrhea till the end of experiment. The problem of the watery consistency of feces was resolved as early as day 6 in the case of groups receiving oral phage-cocktail-containing beads (Group B and C), but the fecal consistency remained watery in the untreated groups (Group D and E) throughout the experiment. Also, the birds of the untreated group were having swelling in the abdominal region, but the birds of group receiving oral phage-cocktail-containing beads appeared to start regaining their vigour and behaved normally from day 6 onwards. Inflammation in the case of the untreated group could be attributed to invasion of intestinal mucosa with high bacterial loads, leading to the stimulation of pro inflammatory cytokines [46].

#### 3.8.2. Gross Pathological Evaluation

**Figure 6:**
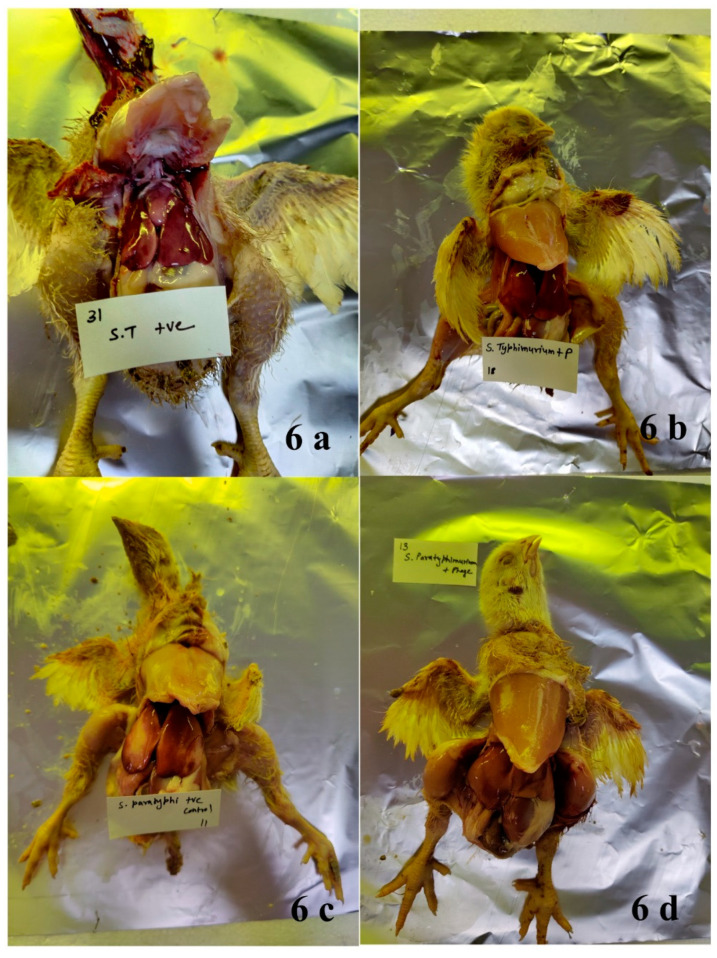
Birds showing severe-to-moderate lesions: (**a**) bird infected with *S. typhimurium* (ST + ve control); (**b**) bird infected with *S. typhimurium* followed by oral treatment with phage-cocktail-encapsulated beads (ST + P); (**c**) bird infected with *S. paratyphi* (SP + ve control); (**d**) bird infected with *S. paratyphi* followed by oral treatment with phage-cocktail-encapsulated beads (SP + P). Severe congestion in the liver of the untreated groups is visible.

To assess the efficacy of the phage delivery system developed by encapsulation in alginate beads and further coating, the birds (three nos. from each group/day 8, 10, 13, 19, and 24, respectively) were sacrificed, and a systemic necropsy was performed immediately after euthanasia. It was revealed that the lesions were specifically limited to the digestive system, involving the liver and the intestine. The birds of different groups showed severe to moderate lesions (Figure 6a–d) post oral infection with *S. typhimurium*/*S. paratyphi*. The symptoms included congestion in the liver and intestine, haemorrhagic streaks in the liver, thickened, enlarged, and fluid-filled caecal tonsils, inflammation in the colon region, and edema in the intestine. The symptoms observed following *Salmonella* infection are primarily due to inflammatory responses elicited by the bacteria’s presence and their toxins [43]. Such symptoms were resolved on the eighth day in the treated groups compared with 14 days in the untreated groups. By day 10, most birds were healthy in the treated groups, while severe lesions were observed in the untreated group. By effectively reducing the bacterial population, phage therapy may decrease the inflammatory response associated with the infection. This can lead to quicker resolution of symptoms such as edema and inflammation in affected tissues. Also, successful phage treatment may help restore normal gut flora more rapidly than untreated infections, aiding recovery from gastrointestinal symptoms [47].

#### 3.8.3. Histopathological Examination

**Figure 7:**
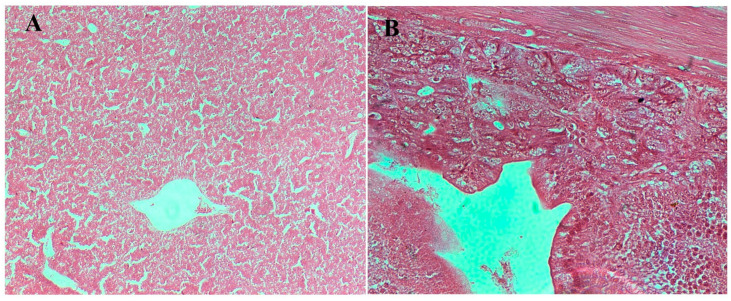
Microscopic examination of tissues from chicks in negative control. (**A**) Section of liver (10×) from chick in negative control showing well-preserved hepatic architecture with no pathological changes and (**B**) Caecum (40×) from chick in negative control with intact mucosal architecture with elongated, slender villi and well-formed crypts.

**Figure 8:**
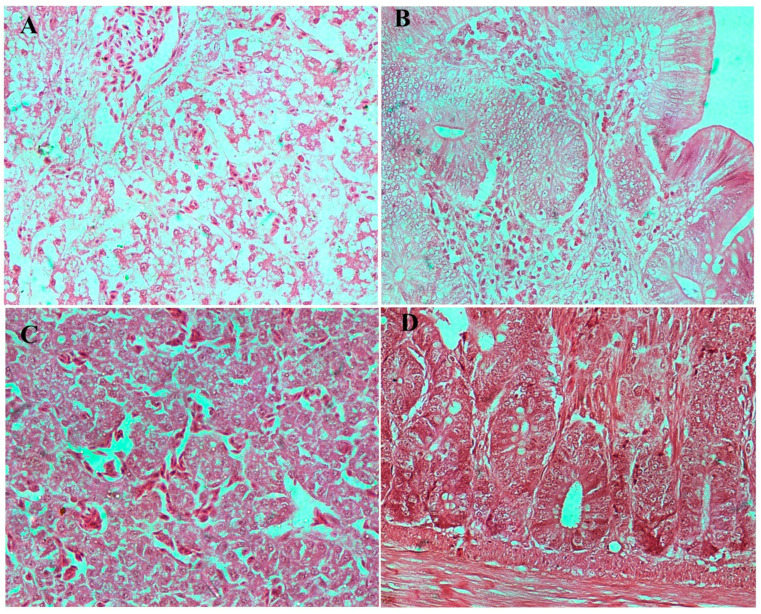
Microscopic examination of tissues from infected birds with *S. paratyphi* before and after treatment with phage-cocktail-encapsulated in alginate beads. (**A**) Section of liver in infected bird showing congested central vein, diffused necrosis of hepatocytes, and infiltration of heterophils in the liver were observed. (**B**) Caecum section of infected bird showing goblet cell hyperplasia, edema, hemorrhages, and heterophilic infiltration. (**C**) Liver section after treatment showing congested central vein, and hemorrhages. (**D**) Caecum section after treatment showing mild goblet cell hyperplasia in the caecum. Images are at 40× magnification.

**Figure 9:**
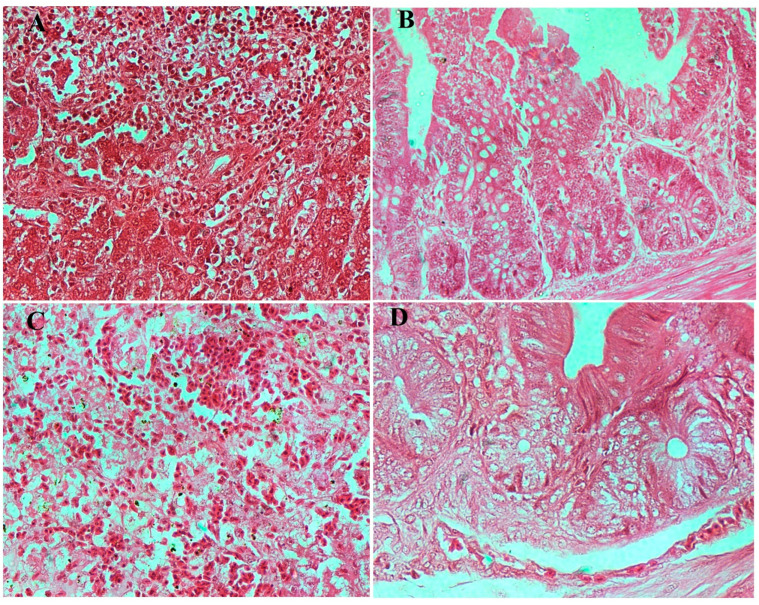
Microscopic examination of tissues from infected birds with *S. typhimurium* before and after treatment with phage-cocktail-encapsulated in alginate beads. (**A**) Section of liver in infected bird showing focal areas of hepatic necrosis, hemorrhages, and lymphocytic infiltration. (**B**) Caecum section of infected bird showing necrosis, desquamation of epithelial lining, and extensive goblet cell hyperplasia. (**C**) Liver section after treatment showing diffused areas of hemorrhages. (**D**) Caecum section after treatment showing mild goblet cell hyperplasia. Images are at 40× magnification.

Microscopic examination of liver and caecum sections revealed notable differences between experimental groups. The negative control (un-infected chicks) showed well-preserved hepatic architecture in liver and intact mucosa in caecum (Figure 7). The infected/no treatment groups showed extensive lesions in the liver, including congestion, extravasation of erythrocytes, focal necrosis, and heterophilic to lymphocytic infiltration. The lesions in the caecum, which is the site of colonization by both bacteria, included goblet cell hyperplasia, edema, necrosis, and desquamation of the epithelial lining, as well as heterophilic infiltration in some instances. The lesions in both the *S. paratyphi* (Figure 8) and *S. typhimurium* (Figure 9) groups given oral treatment of bacteriophage led to a moderate-to-substantial reduction in the intensity of the lesions.

#### 3.8.4. Estimation of Bacterial Load and Phage Presence in Feces and Different Organs

**Figure 10:**
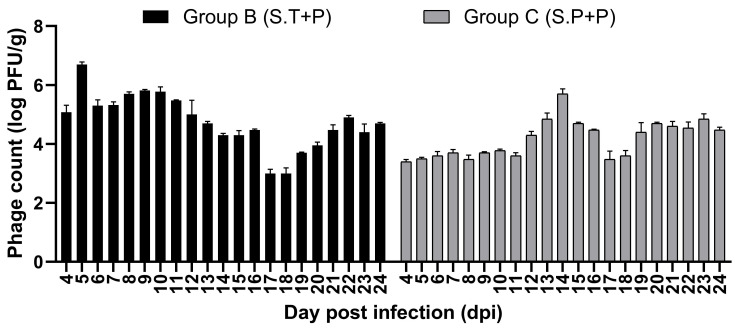
Detection of viable phage particles in feces of treated Groups.

It was observed that phages could be delivered successfully using the PVA-Poly-L-Lysine-coated alginate beads in the gut as the viable phage particles were detected in the feces of the treated groups (Group B and C) up to 24 days of the experiment (Figure 10).

**Figure 11:**
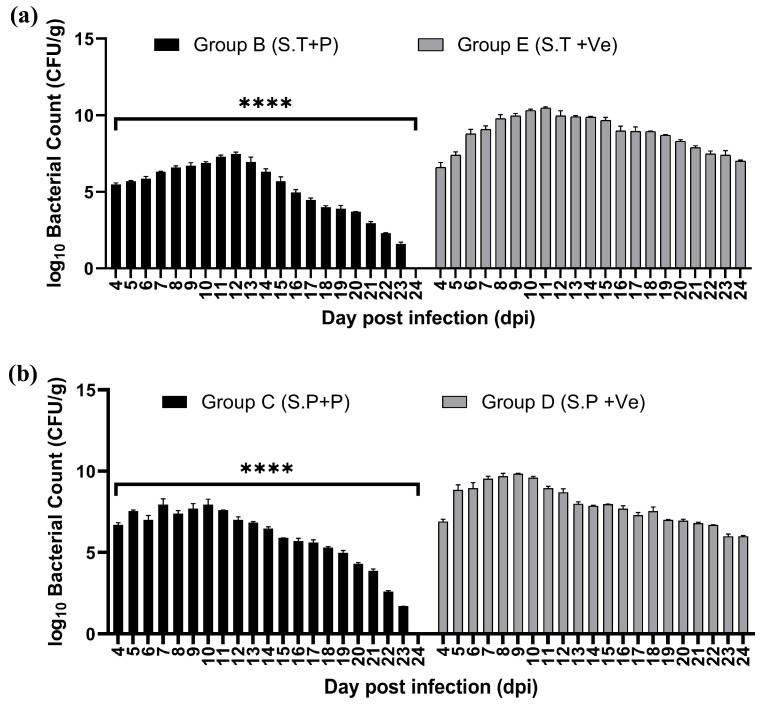
Comparison of fecal bacterial load in treated and untreated poultry groups in case of (**a**) *S. typhimurium*, (**b**) *S. paratyphi*. **** indicates *p* < 0.0001.

The fecal bacterial load was estimated per g wt of poultry litter on various days, and the bacterial load was estimated to be lower in treated groups (Groups B and C) compared with untreated groups (Groups D and E). The difference in the bacterial load in the treated vs. untreated groups (in the case of *Salmonella typhimurium* and *Salmonella paratyphi*) was significantly (****—*p* < 0.0001) reduced on all days of assessment during the experiment (Figure 11a,b). The fecal bacterial load was also eliminated by day 24 post-infection in the treated groups (Groups B and C). However, a high bacterial load had persisted in the feces of untreated groups by day 24 (Figure 11a,b). This reduction is primarily attributed to specific targeting and lysis of bacteria by phages. High specificity of phages, combined with their ability to replicate and lyse target bacteria, makes them a promising tool for controlling bacterial populations [48].

**Figure 12:**
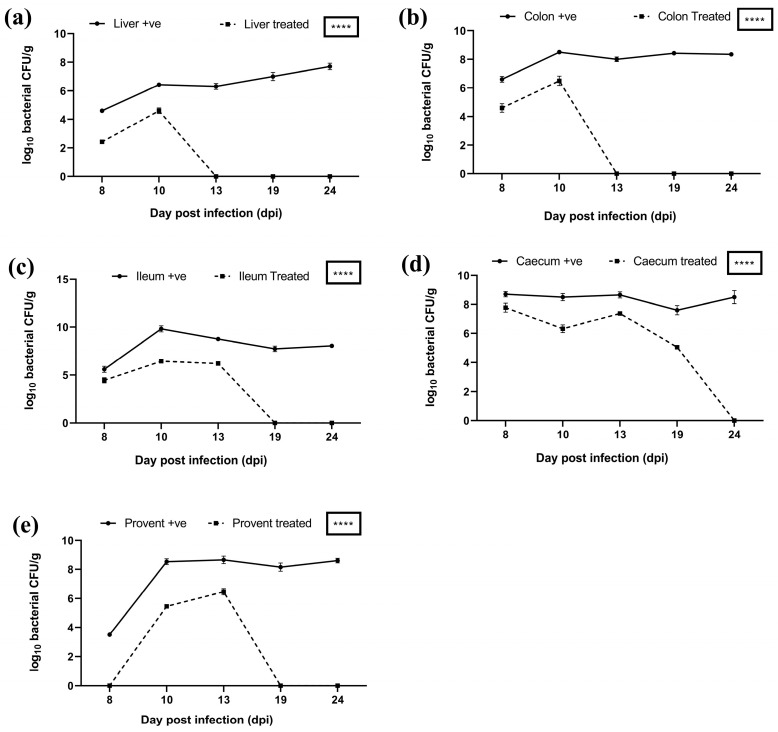
Bacterial load in different organs of treated vs. untreated birds infected with *Salmonella typhimurium* in various organs: (**a**) liver, (**b**) colon, (**c**) ileum, (**d**) caecum, and (**e**) proventriculus. **** indicates *p* < 0.0001.

**Figure 13:**
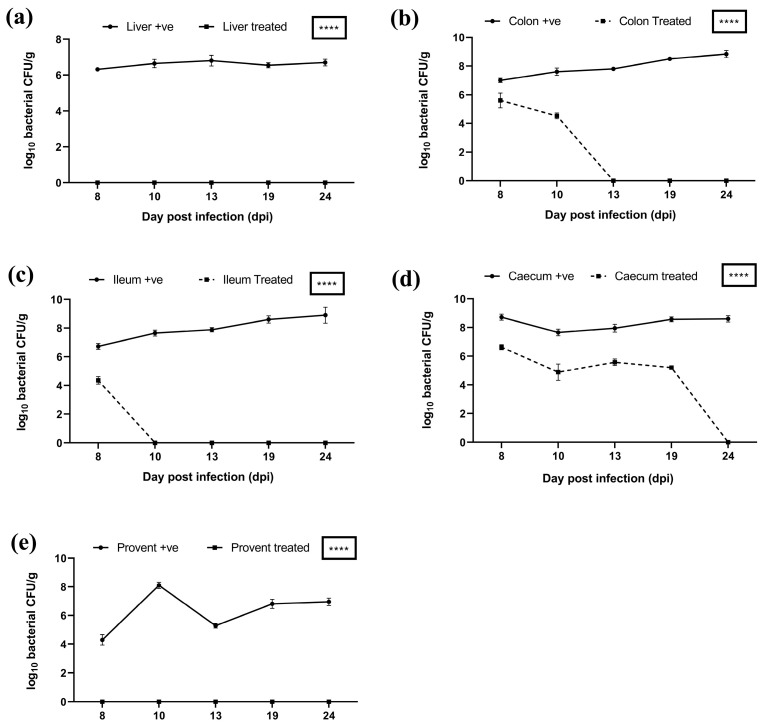
Bacterial load in different organs of treated vs. untreated birds infected with *Salmonella paratyphi* in various organs: (**a**) liver, (**b**) colon, (**c**) ileum, (**d**) caecum, and (**e**) proventriculus. **** indicates *p* < 0.0001.

In the case of both *S. typhimurium* and *S. paratyphi* experimental groups, on days 8 and 10, there was a higher bacterial load in the liver and colon of untreated birds compared to treated birds (Figure 12a,b and Figure 13a,b). On day 13 onwards, the bacterial load was eliminated from the colon in treated birds of both *S. typhimurium* and *S. paratyphi* groups compared with a persistent heavy bacterial load in the colon of untreated birds (Figure 12b,c). In the case of *S. typhimurium* groups, there was a reduced bacterial load in the treated vs. the untreated group up to day 13. Also, the bacterial load was eliminated from the ileum of birds in the treated group in comparison to a heavy bacterial load in the ileum of birds of untreated group on 19 days onwards (Figure 12c). In case of *S. paratyphi* group, on day 8, there was a higher bacterial load in the ileum of birds of untreated vs. treated birds; however, from day 10 onwards, the bacterial load was eliminated from ileum in birds of treated birds in comparison with a heavy bacterial load in the ileum of untreated birds at all successive intervals (Figure 13c).

On days 8, 10, 13, and 19, there was a higher bacterial load in the caecum of untreated birds in comparison with treated birds in cases of both the *S. typhimurium* and *S. paratyphi* (Figure 12d and Figure 13d). On days 8, 10, and 13, there was a higher bacterial load in the proventriculus of untreated birds of the *Salmonella typhimurium* infected group compared to the treatment group. The proventriculus in the case of *S. paratyphi* was found to harbour no bacteria in the treatment group compared to a high bacterial load in the untreated group (Figure 12e and Figure 13e). The bacterial load difference mentioned above was highly significant (****p < 0.0001) in all these groups.

Alleviation of bacterial loads in organs of treated groups and elimination of infection on later days indicates effective delivery of phages to these organs. Previous reports also support the use of phage cocktails to reduce Salmonella [49,50]. Upon the oral administration of alginate beads carrying phage cocktail in the broilers, it was evident that the live phage particles were recovered from poultry litter, and the bacterial load was significantly reduced (*p* < 0.0001) in the intestine of the broilers. This indicates that the encapsulation of phage cocktail in alginate beads, which have been produced by incorporating polyvinyl alcohol (PVA), PVP-K30, and calcium carbonate and have been coated with poly-L-lysine provides an effective mechanism of phage delivery. The method provides an effective formulation to stabilize biologically active phages against highly acidic pH and the action of bile salts.

## 4. Conclusions

Several *in vitro* studies were performed to evaluate the performance of alginate beads as a carrier for the oral delivery of bacteriophage cocktail, such as their ability to protect phages from acidic pH and bile salts while providing a release effect at higher pH. Further evaluation of beads in poultry revealed their potential to deliver a phage cocktail for treating *Salmonella* infection. Phage encapsulation with the alginate beads could ensure targeted delivery of high titres of phages to the caecum, providing encapsulated phages with protection from the harsh environmental conditions in the PV-Gizzard. Furthermore, the complete elimination of infection in the caecum—a known area with elevated *Salmonella* colonization—indicates efficacy of this phage encapsulation method for managing the bacteria in the field. Conversely, the ease with which the phage spreads through excrement might help manage the bacteria in the farming environment. The results of this study nicely demonstrated the protective effect of prepared beads, thus indicating the suitability of the beads for oral delivery.

## Figures and Tables

**Table 1:** Plaque characteristics of isolated phages.

S. No.	Host	Phage	Plaque Characteristics
1	*Salmonella typhimurium*	*Salmonella* phage ΦST143	1.5 mm in diameter, clear, circular
2	*Salmonella enteritidis*	*Salmonella* phage ΦST187	2 mm in diameter, clear, circular
3	*Salmonella enteritidis*	*Salmonella* phage ΦST188	1.5 mm in diameter, clear, circular

**Table 2:** Survival of free and encapsulated bacteriophage after treatments with 1% bile.

Preparation Type	Initial Titre (log_10_ PFU/mL)	Titre After 3 h (log_10_ PFU/mL)
Free phage	9.04	5.60
Encapsulated phage	6.04	5.78

## Data Availability

The bacteriophages are stored, accessioned, and available at Bacteriophage repository, National Centre for Veterinary Type Cultures, ICAR- National Research Centre on Equines, Hisar.
